# Catheter Ablation for Atrial Fibrillation in Breast Cancer Survivors: An Exploratory Observational Study Using the French Nationwide Health Care Database Sample

**DOI:** 10.1002/cnr2.70320

**Published:** 2025-08-25

**Authors:** Laura Saint‐Lary, Juliette Thariat, Baptiste Pinel, Gaëlle Jimenez, Marie‐Odile Bernier, Loïc Panh, Serge Boveda, Sophie Jacob

**Affiliations:** ^1^ Autorité de Sûreté Nucléaire et de Radioprotection (ASNR), PSE‐SANTE/SESANE/LEPID, Laboratoire D'épidémiologie Fontenay‐aux‐Roses France; ^2^ Department of Radiation Oncology Comprehensive Cancer Centre François Baclesse Caen France; ^3^ Department of Radiation Oncology Clinique Pasteur Toulouse France; ^4^ Department of Cardiology and Heart Rhythm Management Clinique Pasteur Toulouse France

**Keywords:** catheter ablation, data claims, epidemiology, radiotherapybreast cancer

## Abstract

**Background:**

Catheter ablation is a key treatment for atrial fibrillation (AF). This procedure is clearly identifiable in French medical‐administrative databases and can be used as a surrogate for symptomatic patients with drug‐refractory or symptomatic paroxysmal AF forms. Breast cancer (BC) patients have an increased risk of AF, but knowledge about AF forms treated by ablation is limited.

**Aims:**

Based on a representative cohort of BC patients from the French National Health Databases, we aimed to assess the occurrence of AF catheter ablation in these patients, compared to the general population.

**Methods:**

Patients with a first diagnosis of BC between 2008 and 2018 and followed for at least 1 year until 2019 were eligible. The incidence rates of AF ablation among BC patients were compared to those in the general population by estimating a standardized incidence ratio (SIR). A competing risk survival model was used to evaluate the occurrence of AF ablation in BC patients treated with or without radiotherapy (RT) (subdistribution Hazard Ratio—sdHR).

**Results:**

We included 3667 patients (78% with RT). Overall, 16 cases underwent AF ablation, whereas 8.3 cases were expected in the general population, resulting in a significant SIR of 1.93 [1.10–3.00] (*p* < 0.05). After a mean follow‐up of 6.6 years, the cumulative incidence of AF ablation at 5 years was 0.14% [0.05–0.35] in the RT group and 0.47% [0.13–1.31] in the non‐RT group, with no significant difference in the age‐adjusted survival analysis (sdHR = 0.65 [0.21–2.01]).

**Conclusions:**

Our exploratory study revealed that BC patients had a twofold greater rate of first AF ablation than the age‐comparable general population, without a link with RT. These results should be interpreted cautiously because of the limited size of the study population and the differing characteristics between the RT and non‐RT groups.

## 1 Introduction

In cardio‐oncology, the risk of atrial fibrillation (AF) has been increasingly explored [1, 2] but evidence in breast cancer (BC) patients is scarce. It has been suggested that BC patients have an increased risk for new onset AF within the first months or the years following BC diagnosis [3], but other studies did not confirm these results [4, 5, 6]. The increased AF risk may be partly attributable to radiotherapy (RT), as observed in studies comparing BC patients treated with RT with the general population or with BC patients not treated with RT [7, 8]. Among AF treatments, catheter ablation is an established therapy for symptomatic patients with drug‐refractoryAF, and as first‐line therapy in selected patients with symptomatic paroxysmalAF, or for patients with heart failure with reduced ejection fraction (HFrEF) to improve ventricular function and cardiovascular outcomes. This procedure is recommended to reduce AF burden, to improve quality of life, and to reduce the risks of cardiovascular outcomes. However, despite the extensive evidence base, there are no specific recommendations for catheter ablation among patients with cancer [9] and no differences in arrhythmia‐free survival following AF catheter ablation in cancer patients treated with or without RT [10]. Howevea r, higher risk of procedural complications of AF ablation in patients with cancwasere reported in a recent meta‐analysis [11]. Consequently, the occurrence of AF ablation could be relevant to explore symptomatic AF forms with drug‐refractory and symptomatic paroxysmal AF forms post‐BC diagnosis compared the to general population. To our knowledge, this study is the first one to explore these AF forms occurrence in the specific BC patients, using the French health insurance database. This study should provide new substantial knowledge about the AF catheter ablation procedre, among frail patients—particularly those with prior RT. In this context, we conducted an observational cohort stuthatich aimed to evaluate the occurrence of AF catheter ablation in BC patients treated with or without RT compared to the general populaton, and to compare incidence of AF catheter ablation in the RT group to the non‐RT group, using the French nationwide health care database sample.

## 2 Materials and Methods

We conducted an exploratory observational study over the period 2008 to 2019, based on the 1/97th representative sample of the French National Health Insurance Claims Database (Echantillon Généraliste des Bénéficiaires, EGB database). The representativeness was confirmed for age, gender, occupation, and medical expenses, but not for region. At the opening in 2006, patients having one randomly assigned checksum number out of the 97 possible numbers in the national health care number and having one reimbursement for a medical care were selected. In the next years, patients who did not receive reimbursement in the previous year were randomly selected as previously explained (1/97th) [12]. This sample provides anonymized data on demographics (gender, year of birth, date of death); long‐term diseases (LTD); reimbursed outpatient healthcare encounters (visits, medical procedures, lab tests, drugs, medical devices); and hospital procedures and discharge diagnoses. Diagnoses identified in LTD and hospital discharges are coded according to the 10th revision of the International Classification of Diseases (ICD‐10), and all medical procedures are coded according to the French Common Classification of Medical Procedures (CCAM).

We selected adult women, aged more than 18 years, affiliated with the general insurance scheme, without a history of BC or FA to constitute the general population. Adult women with a first BC between 2008 and 2018 were included and followed until 2019 [13], if they survived at least 1 year after BC diagnosis. The studied outcome was the first AF catheter ablation performed at least 1 year after BC diagnosis, and we excluded patients with AF ablation history records before BC diagnosis. BC was identified with LTD using the ICD‐10 codes C50 (Malignant neoplasms of breast) or D05 (Carcinoma in situ of breast). The date of BC diagnosis was defined as the date of BC LTD. Surgery, radiotherapy, and chemotherapy were identified using CCAM codes. The outcome, a first AF ablation occurring at least 1 year after BC diagnosis, was identified with medical procedures codes in CCAM (DEPF033, DEPF006, DEPF001, DEPF012, DEPF014, DEPF025, DENF001, DENF003, DENF014, DENF017, DENF018, DENF021). Patients with a history of diabetes were identified by using LTD codes (E10‐E14) or ATC system codes (A10), and those with a history of treated hypertension with ATC system codes (C02).

First, we compared the AF catheter ablation occurrence in the BC cohort relative to the general population, using age and calendar year standardized incidence ratio (SIR). Annual reference rates of AF catheter ablation were calculated for adult women in the general population from the EGB database between 2008 and 2019, by age category (< 40; 40–50, 50–60, 60–70, 70–80; > 80) and calendar period (1 year category). The SIR was obtained by dividing the observed number of AF catheter ablation cases by the expected number of AF catheter ablation cases estimated from general population reference rates. Second, the incidence of AF catheter ablation in patients treated with external beam RT was compared to no‐RT patients using survival analysis accounting for death as a competing risk, to prevent the overestimation of AF ablation occurrence that could happen if it was not accounted for [14]. The cumulative incidence function of AF catheter ablation was estimated for both groups (RT and non‐RT group) and compared with Gray's test. A Fine and Gray subdistribution hazard model was used to examine the association between the time to AF catheter ablation and the BC treatment group (RT and non‐RT group), by estimating subdistribution hazard ratios and their 95% confidence intervals (sd HR). In univariate analysis, the proportional subdistribution hazard regression model was adjusted for available identified risk factors (age, BC treatments including surgery and chemotherapy, diabetes and treated hypertension). Finally, in multivariable analysis, only variables with a *p* value < 0.20 were considered. Analyses were performed using SAS Enterprise Guide, version 4.3, and SAS version 9.4.

## 3 Results

**FIGURE 1:**
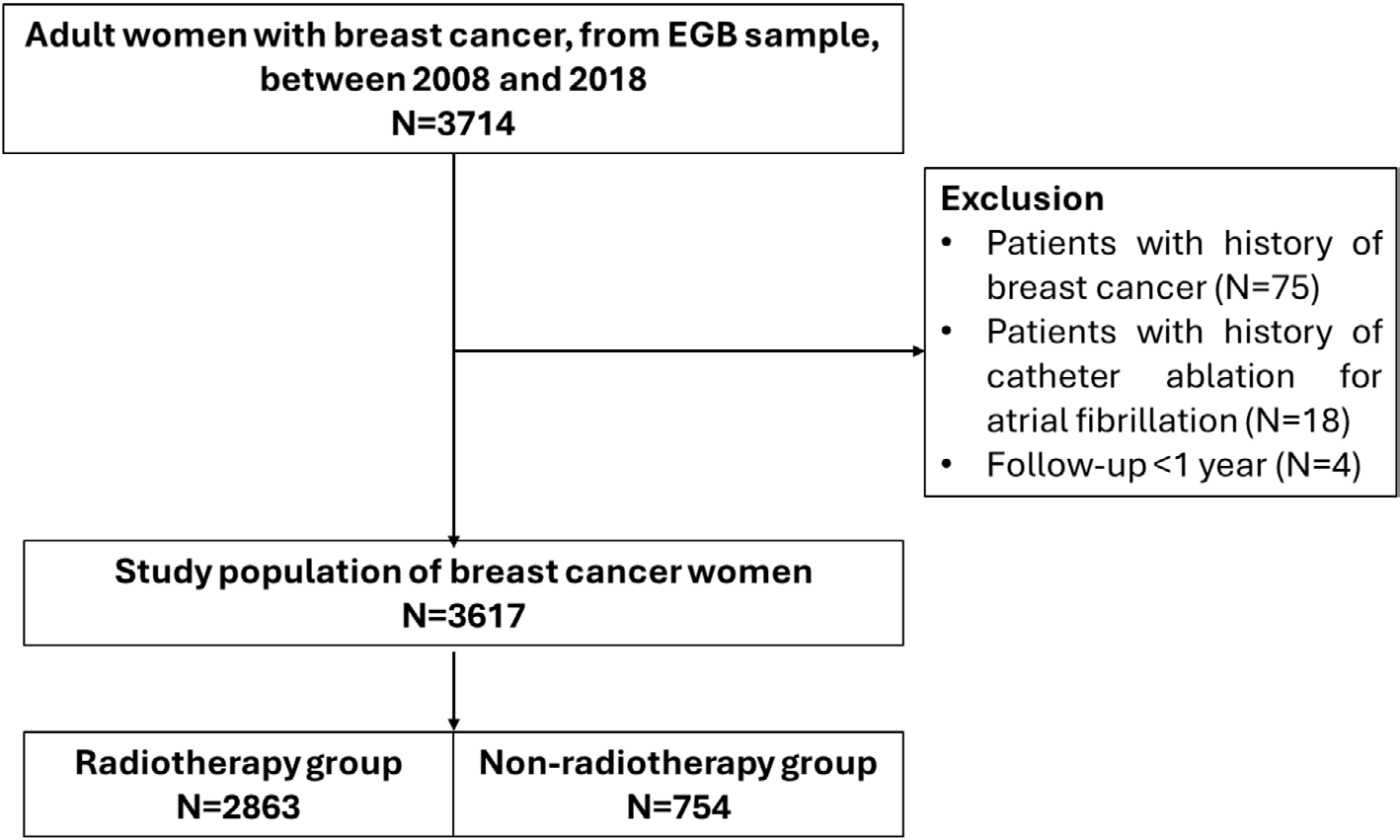
Flow‐chart of the breast cancer cohort study.

**FIGURE 2:**
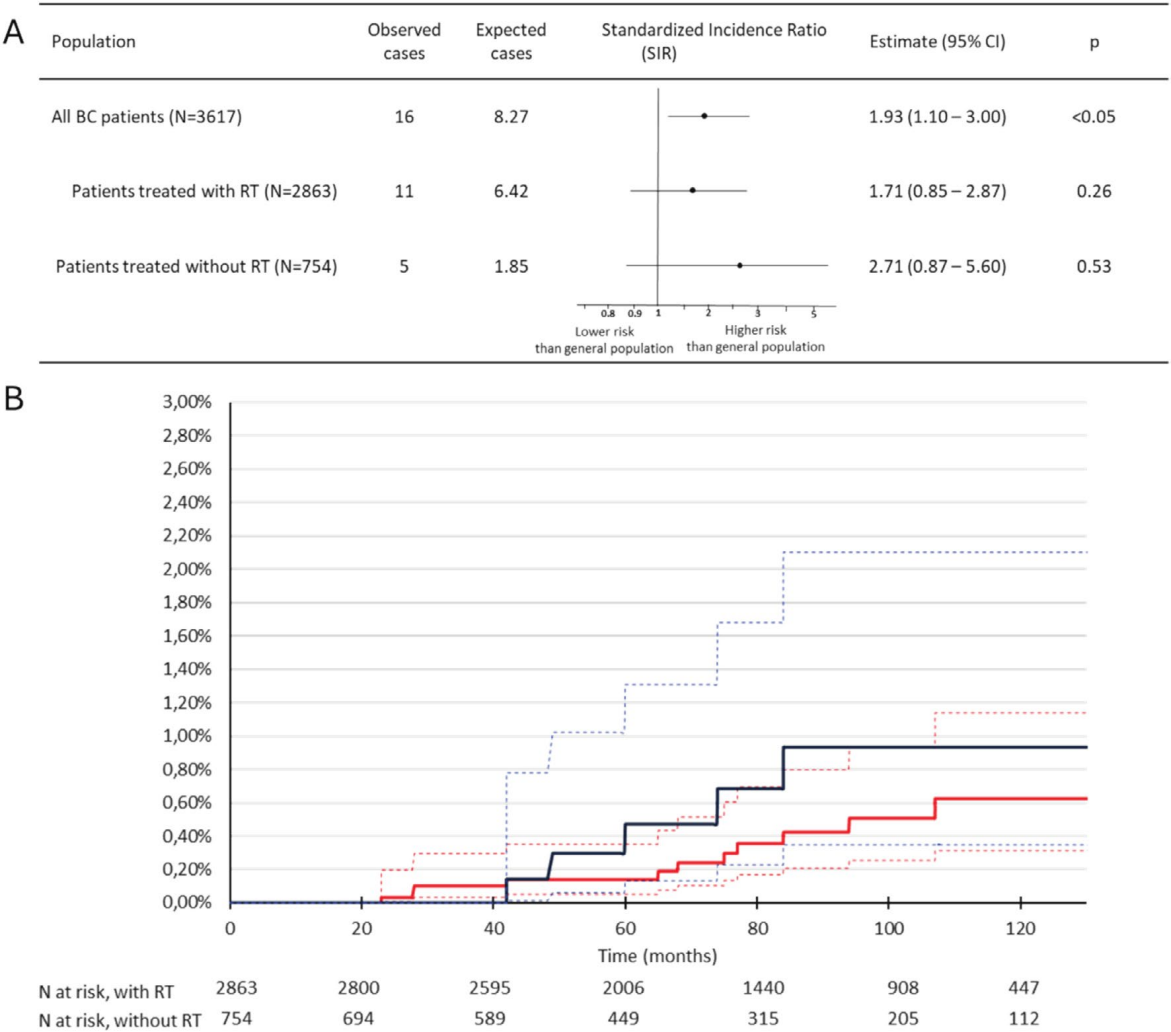
(A) Standardized incidence ratio of AF catheter ablation. (B) Cumulative incidence of AF catheter ablation, according to BC treatment group. (A) Standardized incidence ratio (SIR) standardized on age and calendar year; 95% CI confidence interval; BC breast cancer; observed cases and expected cases of catheter ablation for atrial fibrillation; RT radiotherapy. (B) Red lines for patients treated with RT, blue for patients treated without RT. Dotted lines for 95% confidence interval. *p* value for unadjusted Gray's test is *p* = 0.31.

The study included 211,775 patients in the general population and 3617bc patients between 2008 and 2018, with 2863 (78%) patients treated with RT (Figure 1). Mean age at BC diagnosis was 61 years. Patients treated with RT were younger (59.9 vs. 66.0 years, *p* < 0.01) (Table 1). Mean follow‐up was 6.7 years. Among all BC patients, 16 cases of AF catheter ablation were observed during the follow‐up period from2008 to 2019, as opposed to 8.27 expected cases, corresponding to a significant SIR of 1.93 [95% CI: 1.10–3.00] (*p* < 0.05) (Figure 2A). Subgroup analysis according to treatment with or without RT also revealed higher occurrences than expected in the general population, despite non‐significant: for RT patients, the SIR was 1.71 [95% CI: 0.85–2.87] (*p* = 0.26) based on 11 observed cases, for no‐RT patients, the SIR was 2.71 [95% CI: 0.87–5.60] (*p* = 0.53) based on 5 observed cases. Overall, the 5‐year cumulative AF catheter ablation incidence in BC patients was 0.20% [95% CI: 0.10%–0.60%]. No significant differences were noted between RT patients and non‐RT patients in the 5‐year cumulative AF catheter ablation incidence (0.14% vs. 0.47%, Gray's test *p* = 0.31) (Figure 2B). Age and diabetes were significantly associated with AF catheter ablation occurrence (*p* < 0.05) and were considered in multivariable Fine and Gray survival model that showed non‐significant hazard ratio for AF catheter ablation in the RT‐group compared to no‐RT group (sdHR = 0.65, 95% CI 0.21–2.01; *p* = 0.45) (Table 2).

**TABLE 1:** Baseline characteristics of the 3617 bc patients.

	All BC	RT	Non‐RT	*p*
	*N* = 3617	*N* = 2863	*N* = 754	
Age at BC diagnosis, in years (mean ± SD)	61.2 (13.9)	59.9 (13.1)	66.0 (16.0)	**< 10** ^**−4**^
Surgery *N* (%)	3250 (89.9)	2786 (97.3)	464 (61.5)	**< 10** ^**−4**^
Conservative *N* (%)	2418 (74.4)	2240 (80.4)	178 (38.4)	**< 10** ^**−4**^
Mastectomy *N* (%)	832 (25.6)	546 (19.6)	286 (61.6)	**< 10** ^**−4**^
Chemotherapy, *N* (%)	1068 (29.5)	923 (32.2)	145 (19.2)	**< 10** ^**−4**^
Diabetes *N* (%)	410 (11.3)	308 (10.8)	102 (13.5)	0.03
Treated hypertension *N* (%)	380 (10.5)	291 (10.2)	89 (11.8)	0.19
Death *N* (%)	501 (13.9)	302 (10.6)	199 (26.4)	**< 10** ^**−4**^
AF catheter ablation, *N* (%)	16 (0.44)	11 (0.38)	5 (0.66)	0.30
Follow‐up^a^, in years (mean ± SD)	6.7 (2.8)	6.8 (2.7)	6.1 (3.1)	**< 10** ^**−4**^

Abbreviations: AF: atrial fibrillation; BC: breast cancer; RT: radiation therapy; SD: standard deviation.

^a^ Follow‐up is stopped at AF catheter ablation, or death, or December 31st, 2019.

**TABLE 2:** Analysis of radiotherapy treatment and other risk factors of atrial fibrillation catheter ablation based on Fine and Gray's sub‐distribution hazard model for catheter ablation.

	*N*	Univariate analysis		Multivariate analysis	
		sd HR (95% CI)	*p*	sd HR (95% CI)	*p*
Breast cancer (BC)	3617				
Without radiotherapy	754 (22%)	1		1	
With radiotherapy	2863 (78%)	0.58 [0.20–1.68]	0.32	0.65 [0.21–2.01]	0.45
Age at BC diagnosis	61.2 (13.9)	1.02 [1.00–1.04]	**0.04**	1.01 [0.99–1.04]	0.33
Treated hypertension					
No	3237 (89.5%)	1			
Yes	380 (10.5%)	1.21 [0.27–5.33]	0.80		
Diabetes					
No	3207 (88.7%)	1			
Yes	410 (11.3%)	4.70 [1.71–12.87]	**0.003**	4.32 [1.57–11.88]	**0.005**
Chemotherapy					
No	2549 (70.5%)	1			
Yes	1068 (29.5%)	1.04 [0.36–3.01]	0.94		

Abbreviations: BC breast cancer; RT radiotherapy; sd HR (95% CI): sub‐distribution hazard ratio with 95% confidence.

## 4 Discussion

Our exploratory study revealed that BC patients had a twofold rate of first AF catheter ablation than the age‐comparable general population. Acknowledging the sample‐size limitations, this result is consistent with previous findings on higher AF occurrence in BC patients [15]. Due to our small sample size (*n* = 16 cases of AF ablation) and the induced lack of statistical power, our results suggest a higher but non‐significant rate of AF catheter ablation in BC patients treated with RT than in the general population, as observed for AF occurrence in another study based on electronic hospital records [8]. Moreover, we did not find a significant difference in AF ablation rates between the RT and no‐RT groups. Even if our study focused on the occurrence of AF ablation and not on AF incidence itself, this result is not consistent with results obtained from other studies that reported an association between chest radiotherapy in BC patients and AF incidence [16]. Also, this result should be interpreted cautiously, as non‐RT patients tended to be older, had more comorbidities, and appeared to be in more advanced cancer stages (see Table 1). Indeed, non‐RT patients were less likely to have surgery, and when surgery was performed it was mainly mastectomy, which is indicated when the tumor is large or multiple foci are present [17]. All these factors may independently increase cardiovascular risk, thereby confounding the direct impact of RT. We also acknowledge the potential for procedural referral bias in AF catheter ablation, particularly given the frailty of BC patients—especially those with a history of RT. This bias may have influenced the observed treatment patterns and outcomes. In our subgroup analysis by BC treatment (RT vs. non‐RT), we observed a non‐significant but higher rate of AF catheter ablation among non‐RT patients compared to those who received RT. We faced limitations due to unavailable information in the EGB databases, including absorbed radiation doses and breast cancer laterality, both of which can serve as proxies for cardiac exposure to ionizing radiation during RT. Also, in this exploratory study, other risk factors for AF, such as dyslipidemia, obesity, sleep apnea, heart failure, coronary artery disease/ischemic cardiomyopathy, or hyperthyroidism, were not available due to the closure of the EGB database in 2022. These missing variables are critical, as they may significantly influence subsequent AF risk. To reflect new AF ablation cases and differentiate them from recurrent AF ablation cases, we excluded patients with a history of AF catheter ablation. This approach positions AF catheter ablation as a proxy for symptomatic AF forms with drug‐refractory and symptomatic paroxysmal AF forms, acknowledging that this surrogate limits the ability to capture all AF cases and thus underestimate overall AF. The rate of AF ablation was relatively low in our study (only 0.44%) but it is compatible with findings from another study on the proportion of patients with first‐time catheter ablation (average = 0.50%) [18]. Despite these limits, a strength of the EGB database is based on the representativeness of the general population, in terms of age distribution, which allows us to quantify precisely the expected cases of AF catheter ablation and the SIRs. Another strength of the French Health Insurance Database is its reliability to perform epidemiological studies, although the main purpose was for medico‐administrative uses in the reimbursement of health care [19, 20]. The new French EGB database (ESND) available since 2022 will be an opportunity to repeat the study on a larger sample, including wider AF cases (drug treatments; catheter ablation treatment), with follow‐up now extended to 2023. New analyses that incorporate updated follow‐up data using this expanded dataset are currently underway, and results are expected by 2026.

## 5 Conclusions

In conclusion, while our study suggests a heightened rate of AF catheter ablation in BC patients, further research is warranted to evaluate the safety, efficacy, and outcomes of RT in BC patients, as our study did not yield conclusive findings regarding the impact of this BC treatment. Furthermore, given the substantial health impact of AF on outcomes like stroke, heart failure, and cognitive decline, further investigation into AF risk and management in BC patients is critical.

## Author Contributions

All authors had full access to the data in the study and take responsibility for the integrity of the data and the accuracy of the data analyses. Conceptualization: L.S.‐L. and S.J. Data curation: L.S.‐L. and S.J. Formal analysis: L.S.‐L. and S.J. Funding acquisition: S.J. Investigation: L.S.‐L. and S.J. Methodology: L.S.‐L., M.‐O.B. and S.J. Project administration: S.J.; Resources, S.J. Software: L.S.‐L. and S.J. Supervision: S.J. Validation: S.J. Visualization: L.S.‐L. and S.J. Writing – original draft: L.S.‐L. and S.J. Writing – review and editing: L.S.‐L., J.T., B.P., M.‐O.B., S.B. and S.J.

## Ethics Statement

The authors have nothing to report.

## Consent

The authors have nothing to report.

## Conflicts of Interest

The authors declare no conflicts of interest.

## Data Availability

Data were obtained from the French National Health Insurance Claims Database (Echantillon Généraliste des Bénéficiaires, EGB database).
